# Regenerative Therapy for Liver Cirrhosis Based on Intrahepatic Arterial Infusion of Autologous Subcutaneous Adipose Tissue-Derived Regenerative (Stem) Cells: Protocol for a Confirmatory Multicenter Uncontrolled Clinical Trial

**DOI:** 10.2196/17904

**Published:** 2020-03-31

**Authors:** Yoshio Sakai, Shinya Fukunishi, Masayuki Takamura, Oto Inoue, Shinichiro Takashima, Soichiro Usui, Akihiro Seki, Alessandro Nasti, Tuyen Thuy Bich Ho, Kazunori Kawaguchi, Akira Asai, Yusuke Tsuchimoto, Taro Yamashita, Tatsuya Yamashita, Eishiro Mizukoshi, Masao Honda, Yasuhito Imai, Kenichi Yoshimura, Toshinori Murayama, Takashi Wada, Kenichi Harada, Kazuhide Higuchi, Shuichi Kaneko

**Affiliations:** 1 Department of Gastroenterology Kanazawa University Hospital Kanazawa Japan; 2 Department of Gastroenterology Osaka Medical College Takatsuki Japan; 3 Department of Cardiovascular Medicine Kanazawa University Hospital Kanazawa Japan; 4 System Biology Kanazawa University Kanazawa Japan; 5 Department of General Medicine Kanazawa University Hospital Kanazawa Japan; 6 Innovative Clinical Research Center Kanazawa University Kanazawa Japan; 7 Center for Integrated Medical Research Hiroshima University Hospital Hiroshima Japan; 8 Department of Nephrology Kanazawa University Hospital Kanazawa Japan; 9 Department of Human Pathology Kanazawa University Graduate School of Medicine Kanazawa Japan

**Keywords:** adipose tissue-derived regenerative (stem) cells, stromal cells, stem cells, liver cirrhosis, investigator-initiated clinical trial protocol, adipose tissue dissociation device, protocol, stem cell therapy, liver

## Abstract

**Background:**

Liver cirrhosis results from chronic hepatitis, and is characterized by advanced fibrosis due to long-term hepatic inflammation. Cirrhosis ultimately leads to manifestations of jaundice, ascites, and encephalopathy, and increases the risk of hepatocellular carcinoma. Once cirrhosis is established, resulting in hepatic failure, no effective treatment is available. Therefore, novel therapies to inhibit disease progression of cirrhosis are needed.

**Objective:**

The objective of this investigator-initiated clinical trial is to assess the safety and efficacy of autologous adipose tissue-derived regenerative (stem) cell therapy delivered to the liver via the hepatic artery in patients with liver cirrhosis.

**Methods:**

Through consultation with the Japan Pharmaceuticals and Medical Devices Agency, we designed a clinical trial to assess a therapy for liver cirrhosis based on autologous adipose tissue-derived regenerative (stem) cells, which are extracted using an adipose tissue dissociation device. The primary endpoints of the trial are the serum albumin concentration, prothrombin activity, harmful events, and device malfunction.

**Results:**

Enrollment and registration were initiated in November 2017, and the follow-up period ended in November 2019. Data analysis and the clinical study report will be completed by the end of March 2020.

**Conclusions:**

Completion of this clinical trial, including data analysis, will provide data on the safety and efficacy of this novel liver repair therapy based on autologous adipose tissue-derived regenerative (stem) cells using an adipose tissue dissociation device.

**Trial Registration:**

UMIN Clinical Trials Registry UMIN000022601; https://tinyurl.com/w9uqw3q

**International Registered Report Identifier (IRRID):**

DERR1-10.2196/17904

## Introduction

### Background

Liver cirrhosis is the eventual outcome of chronic liver diseases, including chronic hepatitis, autoimmune hepatitis, primary biliary cholangitis, alcoholic hepatitis, and nonalcoholic steatohepatitis (NASH), which is currently increasing in prevalence relative to other liver conditions and is associated with metabolic disease [1,2]. Liver cirrhosis leads to various complications, including esophageal varix, hepatic encephalopathy, ascites, jaundice, and hepatocellular carcinoma, which result in a worse prognosis for patients with cirrhosis [3]. The end stage of cirrhosis is hepatic failure, for which liver transplantation is the only treatment as an extremely invasive procedure that requires continuous immunosuppressive therapy for the remainder of the patient’s life. Moreover, there are very few cadaveric donors in Japan, with only 69 cadaveric donors (and 347 living donors) for liver transplantation available in 2017 [4]. Therefore, novel treatments for cirrhosis should be explored.

Fatty liver disease and NASH are emerging chronic liver diseases. Although steatohepatitis is strongly correlated with metabolic syndrome, it is not clear why steatosis of the liver causes chronic inflammation and ultimately leads to cirrhosis [5].

### Objectives

Mesenchymal stem cells are somatic pluripotent stem cells that can differentiate into various cell types [6,7] and have immunomodulatory capabilities [8]. Freshly isolated adipose tissue is a rich source of mesenchymal stem cells [9]. Similar to bone marrow cells, autologous mesenchymal cells can be used therapeutically. Therefore, freshly isolated autologous adipose tissue-derived stromal cells have attracted substantial attention for potential therapeutic use in various organs [10]. We previously conducted a clinical study to confirm the safety of a liver cirrhosis therapy based on adipose tissue-derived stromal cells [11]. To determine the practicality of this treatment strategy, we designed an open-label, uncontrolled multi-institutional clinical trial for a regenerative therapy for liver cirrhosis based on adipose tissue-derived regenerative (stem) cells (ADRCs). This trial was designed to assess the safety and efficacy of the treatment in consultation with the Japan Pharmaceuticals and Medical Devices Agency (PMDA). Herein, we describe the detailed protocol for the trial.

## Methods

### Study Objectives

The objective of the study is to assess the efficacy and safety of an autologous ADRC therapy delivered to the liver via the hepatic artery.

### Study Design and Enrollment

**Figure 1:**
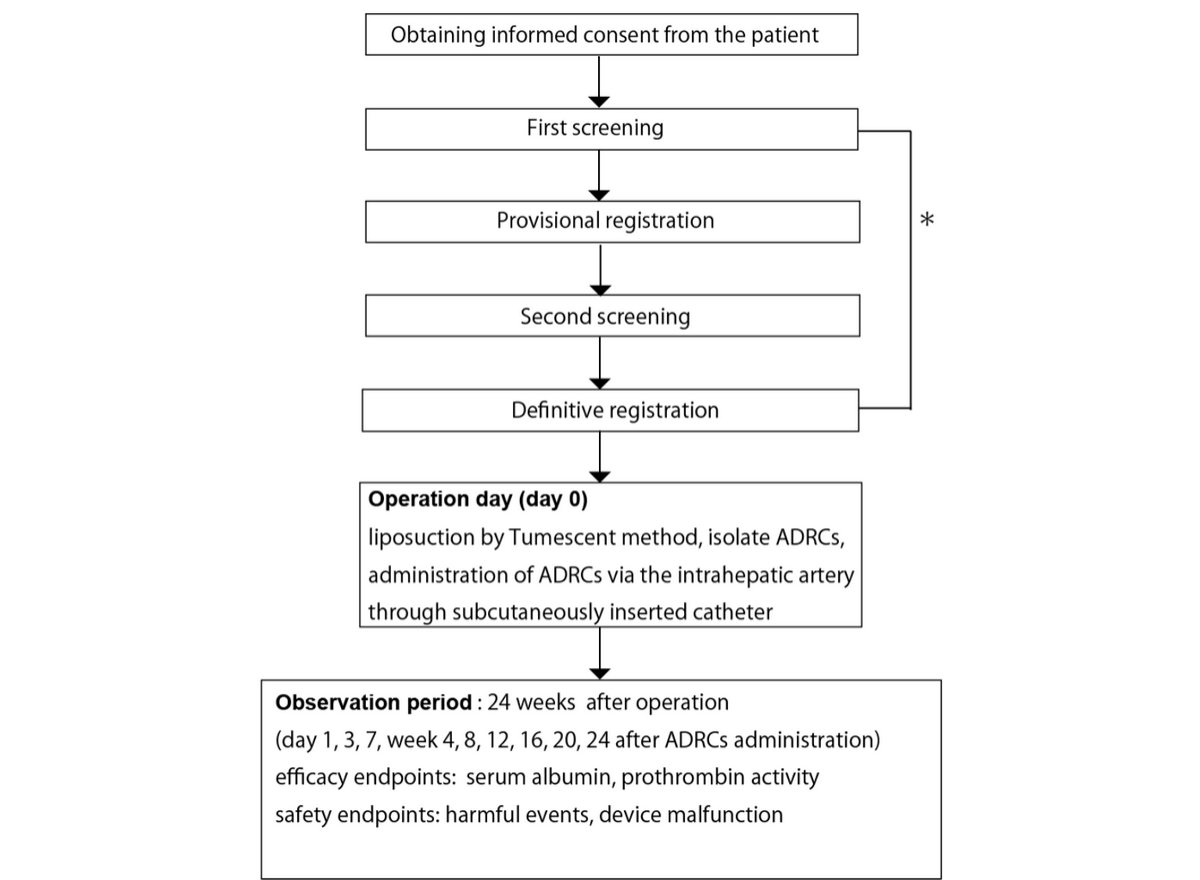
Case enrollment procedure. This trial will enroll patients with nonalcoholic steatohepatitis or fatty liver disease who provided written informed consent. Regarding the steps indicated by the asterisk, screening and registration involve the following: nonalcoholic steatohepatitis patients who drink ≤20 g alcohol per day will simultaneously undergo the first and second screening stages, as well as provisional and definitive registration. Patients who drink >20 g but ≤70 g alcohol will undergo the initial screening and be provisionally registered. These patients are expected to restrict their alcohol consumption to ≤20 g per day for 8 weeks and then undergo the second screening stage. Patients meeting the enrollment criteria at this stage will be definitively registered to undergo the treatment described in this protocol followed by observation for 24 weeks after treatment. ADRCs: adipose tissue-derived regenerative (stem) cells.

This is a multicenter, collaborative, nonblinded, uncontrolled clinical trial carried out in Japan (Kanazawa University Hospital, Osaka Medical College Hospital). The adipose tissue dissociation device used to isolate ADRCs is the investigational trial device. All patients who meet the eligibility criteria and provide informed consent will be enrolled (Figure 1). Multimedia Appendix 1 shows the detailed schedule of the clinical study.

### Eligibility Criteria

#### Inclusion Criteria

Patients who meet all of the following criteria are eligible for the study. A diagnosis of liver cirrhosis is obtained based on imaging or histological examination due to two types of etiology: NASH or fatty liver. NASH is determined according to the following criteria: alcohol intake ≤20 g/day, no other obvious liver injury etiologies, and association with complications such as obesity (especially visceral fat), metabolic syndrome, and diabetes mellitus. Fatty liver disease is determined according to the following criteria [12,13]: alcohol intake >20 g/day but ≤70 g/day (note that the criterion for the amount of alcohol consumed is the same for men and women in accordance with previous publications [12,13]; although the duration of alcohol drinking is not specified, most hepatologists assume that the patient has consumed the defined amount of alcohol for more than 5 years); no other obvious liver injury etiologies or association of complications such as obesity (especially visceral fat), metabolic syndrome, and diabetes mellitus; and ability to maintain alcohol intake at ≤20 g/day from the provisional registration until the second screening (≥8 weeks). Other eligibility criteria include age at the time of providing consent ≥20 years or older but <80 years, total bilirubin concentration ≤3.0 mg/dL at screening, platelet count ≥5.0×10^4^/µL at screening, prothrombin activity ≥70% at screening, serum creatinine level ≤1.5 mg/dL at screening, serum albumin ≤4.0 g/dL at screening, and patients able to provide written informed consent themselves.

#### Exclusion Criteria

Patients are excluded if they meet any of the following conditions: presence of severe pulmonary hypertension such as esophageal gastric varix, which poses a risk of rupture; presence of severe heart diseases, renal diseases, respiratory diseases, blood diseases, blood coagulation disorders, or other serious complications; presence or presumed presence of severe infectious diseases requiring intravenous treatment with antibiotics, antimycotics, or antiviral agents; presence of concurrent malignancy or history of malignancy within the past 5 years not deemed to be fully cured; history of cerebrovascular disease (eg, cerebral infarction, cerebral hemorrhage); known or suspected pregnancy; active treatment with adrenal corticosteroids; inability to maintain alcohol intake at <20 g/day after providing informed consent and for 24 weeks after treatment or discontinuation; treatment with other cell therapies within 6 months before providing informed consent; enrollment in other clinical trials; presumed life expectancy <1 year at the time of providing informed consent; allergy to drugs (eg, anesthetics) used in the trial; history of taking prohibited concomitant medications (antiplatelet drugs, anticoagulant drugs) within 7 days before providing informed consent; and judged to be inappropriate for enrollment by the principal investigator or subinvestigator.

### Treatment

Subcutaneous adipose tissue from the abdomen or buttock of the patient will be obtained by Tumescent liposuction, a standard cosmetic surgery method, under general anesthesia or local/lumbar spine local anesthesia. Regenerative (stem) cells will be isolated using the adipose tissue dissociation system Celution 800/IV (Cytori Therapeutics, San Diego, CA, USA), followed by measurements of cell number and viability in a collected cell aliquot using a nucleocounter (ChemoMetec, Gydevang, Denmark). Cell viability should be at least 70%. Cell aliquots will be prepared at a density of 1×10^6^/mL in Ringer’s lactate. ADRCs, at a density of 3.3×10^5^ cells/kg, will be administered through the common hepatic artery via the tip of microcatheter IV (Asahi Intec Co Ltd, Seto, Aichi, Japan), inserted subcutaneously into a femoral or upper arm artery. For 24 weeks after providing informed consent, albumin products, antiplatelets, and anticoagulants will be prohibited. The following drugs can be continued only when administered after informed consent has been provided: branched-chain amino acids, vitamin E, other nutritional therapies, hepatic protection drugs, and antihyperlipidemia drugs.

### Image Analysis of Chronic Liver Disease and Liver Biopsy

Abdominal computed tomography or magnetic resonance images of the liver will be obtained using a contrast reagent before registration and 24 weeks after treatment. These images will be analyzed by a radiologist and hepatologist who will assess the morphological changes in the liver, spleen, and vasculature due to chronic liver disease. The pathology will be assessed using the nonalcoholic fatty liver disease activity score, which covers steatosis, lobular inflammation, hepatocellular ballooning, and fibrosis [14], in addition to the Matteoni classification [15].

### Follow up

The observation period after treatment will be 24 months during which examinations will be performed at various time points (Multimedia Appendix 1).

### Endpoints

Efficacy endpoints are the serum albumin level and prothrombin activity. The safety endpoints are adverse reactions and device malfunction.

### Sample Size

The serum albumin level is considered a key efficacy endpoint with respect to the proof of concept and mode of action of this treatment. In an earlier clinical study, we observed that 3 of 4 cirrhotic patients who underwent the same treatment as applied in this clinical trial had improved serum albumin levels after 3-6 months [11]. A sample size of 7 patients provided a power of over 85% with a one-sided significance level of .05 to reject the null rate of 3% under the expectation of 40%. This clinical trial was designed to explore safety and efficacy, and therefore a total of 8 patients was considered to be sufficient for this purpose.

### Statistical Analysis

A detailed statistical analysis of efficacy will be performed, and a per-protocol analysis will also be conducted secondarily. Subjects who underwent treatment will be used for the safety analysis. The statistical analysis protocol will be designed before data confirmation and fixation.

### Regulatory Compliance

Approval of this clinical trial was obtained from the institutional review boards of Kanazawa University Hospital and Osaka Medical College Hospital. The study has been registered in the Japanese UMIN Clinical Trials Registry (UMIN000022601). This investigator-initiated clinical trial design was discussed with, and submitted to, the Japan PMDA before enrollment commenced. All treatment procedures in this trial will be carried out in accordance with the Declaration of Helsinki and International Conference on Harmonization Good Clinical Practice guidelines [16]. Written informed consent will be obtained from the patients before the first and second screenings as well as at registration.

## Results

The study is supported by the Japan Agency of Medical Research and Development. The required number of patients has been registered. The data analysis and study report will be completed in March 2020.

## Discussion

Liver cirrhosis is a serious condition that results from chronic liver diseases [1]. Unless the cause of chronic liver disease can be addressed, liver cirrhosis typically worsens, leading to complications such as esophageal varix, ascites, jaundice, and hepatic encephalopathy. Thus, preventing the progression of liver cirrhosis is extremely important for maintaining quality of life and improving the prognosis of patients with cirrhosis.

Mesenchymal stem cells are somatic stromal stem cells that can differentiate into mesodermal lineage cells, adipocytes, osteocytes, and chondrocytes, as well as hepatocytes of the endodermal lineage. In addition, mesenchymal stem cells have immunomodulatory characteristics [8,17], which makes them a promising cell source for treating organ injury associated with inflammation. In this context, many nonclinical and clinical studies have investigated the application of mesenchymal stem cells in regenerative therapy for diseases of various organs [18]. We previously reported the use of murine adipose tissue-derived mesenchymal stem cells to repair murine liver cirrhosis caused by NASH, demonstrating enhancement of albumin production in hepatic parenchymal cells as well as suppression of hepatic inflammation [7]. In addition, we found that freshly isolated stromal vascular cells of the adipose tissue contained a fraction that was immunosuppressive to hepatitis of mice [10]. Thus, ADRCs are considered to be potentially useful for treatment of cirrhosis, contributing to repairing/regenerating the damaged liver.

Treatment procedures involve aspiration of the patient’s own subcutaneous adipose tissue [19,20], isolation of stromal cells from the obtained adipose tissue, and administration of the stromal cells into the cirrhotic liver via the hepatic artery using a catheter. As per our PMDA consultation, this clinical trial of cirrhosis will employ an adipose tissue dissociation system that automatically isolates ADRCs. The benefit of this approach is that it does not involve a complex cell manufacturing process requiring good manufacturing and clinical practice. In addition, the therapy is autologous, which may help avoid unexpected harmful intense immune responses such as allergies.

Chronic liver diseases have various antecedents [21]. Most chronic liver diseases are associated with hepatitis B or C virus infection. Recently, direct-acting antivirals have been developed to treat hepatitis C virus infection and nucleotide analogues are available for the treatment of hepatitis B virus infection. With the former treatment, almost all patients achieve complete elimination of hepatitis C virus [22]. Although fibrosis is not always ameliorated and recovery of the impaired hepatic reserve is gradual [23], the population of hepatitis C virus-infected patients is decreasing rapidly [24]. Nucleotide analogues for hepatitis B are extremely effective at inhibiting virus replication, thus attenuating chronic hepatitis activity. Although the pathogenic mechanisms underlying autoimmune hepatitis and primary biliary cholangitis are not fully understood, they are believed to be related to immune disorders; autoimmune hepatitis responds to steroids and immunosuppressants [25] and primary biliary cholangitis responds to ursodeoxycholic acid [26]. By contrast, there is no established treatment for steatohepatitis.

If the results of this clinical trial are satisfactory and lead to approval of this novel regenerative therapy, a practical treatment will be available for liver cirrhosis that is especially beneficial for patients with steatohepatitis, for which there is currently no effective treatment.

## Appendix

Multimedia Appendix 1 Trial design overview.

## Abbreviations

ADRCs: adipose tissue-derived regenerative (stem) cells
NASH: non-alcoholic steatohepatitis
PMDA: Japan Pharmaceuticals and Medical Devices Agency
